# Use of diamond sensors for a high-flux, high-rate X-ray pass-through diagnostic

**DOI:** 10.1107/S1600577522003022

**Published:** 2022-04-25

**Authors:** J. Bohon, E. Gonzalez, C. Grace, C. T. Harris, B. Jacobsen, S. Kachiguine, D. Kim, J. MacArthur, F. Martinez-McKinney, S. Mazza, M. Nizam, N. Norvell, R. Padilla, E. Potter, T. Prakash, E. Prebys, E. Ryan, B. A. Schumm, J. Smedley, D. Stuart, M. Tarka, I. S. Torrecilla, M. Wilder, D. Zhu

**Affiliations:** a Los Alamos National Laboratory, Los Alamos, NM 87545, USA; b Santa Cruz Institute for Particle Physics, University of California, Santa Cruz, CA 95064, USA; c Lawrence Berkeley National Laboratory, Berkeley, CA 94720, USA; d Sandia National Laboratories, Albuquerque, NM 87123, USA; e SLAC National Accelerator Laboratory, Menlo Park, CA 94025, USA; f University of California, Davis, CA 95616, USA; g University of California, Santa Barbara, CA 93106, USA

**Keywords:** diamond sensors, high-rate diagnostics, high-flux diagnostics

## Abstract

X-ray free-electron lasers (XFELs) deliver pulses of coherent X-rays on the femtosecond time scale, with potentially high repetition rates. While XFELs provide high peak intensities, both the intensity and the centroid of the beam fluctuate strongly on a pulse-to-pulse basis, motivating high-rate beam diagnostics that operate over a large dynamic range. The fast drift velocity, low X-ray absorption and high radiation tolerance properties of chemical vapour deposition diamonds make these crystals a promising candidate material for developing a fast (multi-GHz) pass-through diagnostic for the next generation of XFELs. A new approach to the design of a diamond sensor signal path is presented, along with associated characterization studies performed in the XPP endstation of the LINAC Coherent Light Source (LCLS) at SLAC. Qualitative charge collection profiles (collected charge versus time) are presented and compared with those from a commercially available detector. Quantitative results on the charge collection efficiency and signal collection times are presented over a range of approximately four orders of magnitude in the generated electron–hole plasma density.

## Abstract 2

Two approaches to the design of a diamond sensor signal path were explored using high-intensity X-ray pulses from the LINAC Coherent Light Source at SLAC. Results on the charge-collection efficiency and signal collection time are presented over a range of approximately four orders of magnitude in the generated electron–hole plasma density.

## 1. Introduction

X-ray free-electron lasers (XFELs) are renowned for their capability to provide high-intensity (mJ scale) pulses of coherent X-rays. However, due to the inherently unstable nature of the underlying lasing process, XFEL beams tend to vary widely in intensity and centroid location from pulse to pulse. At the same time, multi-GHz repetition rates, either through pulse splitting and delay (Decker *et al.*, 2010 ▸) or through rapid generation of the full-intensity pulses themselves (Garnett, 2016 ▸), is either currently in use or envisioned for future XFEL facilities. Thus, the development of high-rate beam diagnostics that can be sensitive to generated signal sizes over a large dynamic range are motivated by experiments that can take advantage of knowledge of the intensity and centroid location of each delivered pulse.

Monocrystalline diamond exhibits a number of properties that make it an attractive option for a broad range of sensor applications. Superior radiation tolerance, a fast saturated drift velocity (approximately 200 µm ns^−1^) (Wort & Balmer, 2008 ▸) and superior thermal conductivity (2200 W m^−1^ K^−1^) distinguish diamond among other semiconductor sensor materials, such as silicon and gallium-arsenide, as being particularly suitable for advanced accelerator diagnostics. To establish a general scale, propagating at the saturated drift velocity, charges induced from X-ray absorption will be collected within 2.5 ns for a 500 µm-thick diamond sensor (a thickness readily available from commercial vendors); if thinned to 30 µm, the collection time would be reduced to approximately 150 ps.

Here, we explore the use of diamond sensors as a pass-through diagnostic for XFEL beams. For this application, involving intense X-ray beams that are trained directly on the diagnostic sensor, diamond properties that might be a disadvantage for other applications provide additional advantages relative to other sensor materials. The low atomic number of carbon leads to a relatively small scattering cross section for X-rays above the carbon *K*-shell edge of 0.28 keV, limiting the absorption of the XFEL beam as it passes through the diagnostic. In addition, the large diamond band gap of 5.5 eV, and the resulting pair excitation energy of 13.3 eV (Keister & Smedley, 2009 ▸), limits the production of signal charge relative to other sensor materials; this is advantageous when contemplating a pass-through system for intense X-ray beams, which liberate a surfeit of signal charge.

In this study, we explored the characteristics of diamond-sensor charge collection in limits relevant to their application as pass-through diagnostics for high-intensity, high repetition rate X-ray beams. These studies made use of a readout scheme designed specifically for the treatment of large, high-bandwidth signals, and were performed at the XPP beamline of the LINAC Coherent Light Source (LCLS) at the SLAC National Accelerator Laboratory on 5–6 April 2021. The studies made use of a monochromatic beam of 11.89 keV X-rays with individual pulse varying in energy from 1 µJ to nearly 100 µJ. Both the duration and the efficiency of charge collection were studied as functions of the density of generated charge within the diamond sensor and the magnitude of the applied drift field. A qualitative comparison was also made to the response of a commercially available diamond sensor system, based on data collected on the XPP beamline on 14–15 November 2020, making use of 9.83 keV X-ray pulses in a similar intensity range.

## 2. Sensors and readout schemes

**Figure 1:**
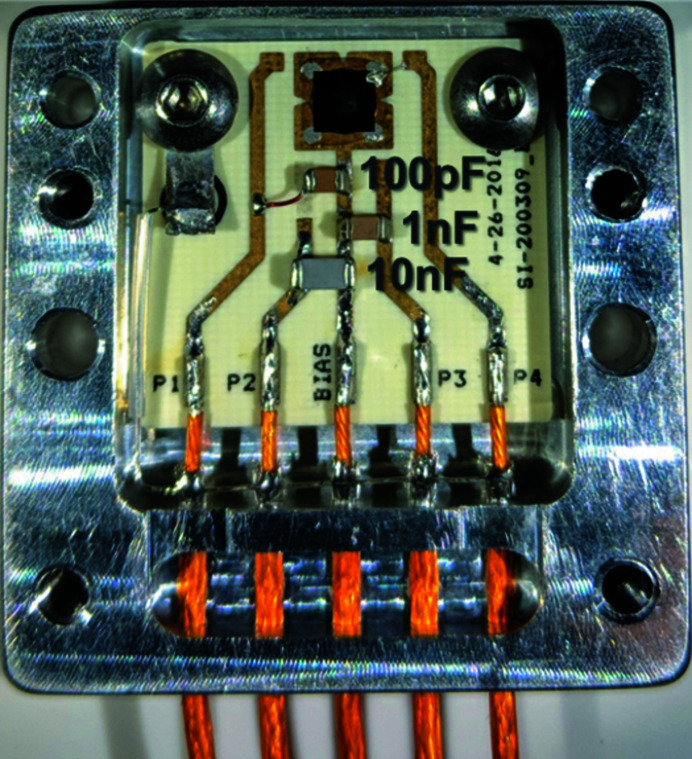
Photograph of the Sydor assembly, with the upper shielding cover removed. The signal readout trace is at the right (P4) and the supply bypass through the 10 nF capacitor was established by connecting trace P2 to ground through the attached RG316 coaxial cable. The sensor is at the top-centre of the assembly and is connected to the readout trace via an array of wire bonds.

The commercial sensor module, exposed in the November 2020 LCLS run, and for which only qualitative response characteristics were explored, was the enclosure from the M405V Position Monitor Module, designed and manufactured by Sydor Technologies (Fairport, NY, USA). The module makes use of a simple readout path, for which the bias voltage supply is tied directly to the sensor, with the opposite side of the sensor connected to the central conductor of an MCA-terminated RG316 coaxial cable. The signal is returned to ground through a data acquisition module, typically an oscilloscope, digitizer or electrometer, with a 50 Ω input impedance. The assembly, referred to below as the ‘Sydor assembly’, was loaded with a 4 mm × 4 mm, 500 µm-thick monocrystalline diamond provided by the Element Six corporation, and was inspired and informed by a similar diagnostic tested at the Paul Scherrer Institute Bernina beamline (Juranic *et al.*, 2019 ▸). Electrodes of approximately 3.5 mm × 3.5 mm, 25 nm-thick platinum were deposited on the diamond substrate at the Center for Integrated Nanotechnologies (CINT) facility in Albuquerque, New Mexico, USA. At the Santa Cruz Institute for Particle Physics (SCIPP) laboratory on the UC Santa Cruz campus, the sensor was affixed to the bias pad with conductive silver ep­oxy, and the readout electrode was bonded to the readout trace via 15 parallel aluminium wire bonds. Relative to the assembly purchased from Sydor Technologies, however, the signal path was augmented, again at the SCIPP laboratory, with a 10 nF capacitor that provided a low-impedance AC signal return path that bypassed the bias voltage power supply. Upon biasing the sensor, this provided a reservoir of charge in the microcoulomb range, available for immediate conduction around the signal path, avoiding the sluggish response of the supply. A photograph of the loaded Sydor assembly is shown in Fig. 1 ▸.

Concerned that the lengthy signal path might present significant impedance to the large, high-bandwidth signals produced by the diamond sensor, a second sensor assembly was designed and assembled at SCIPP. This assembly features a low-impedance signal path, implemented on a printed circuit board (PCB), designed to circulate large amounts of signal charge at high bandwidth. It was loaded with an Element Six monocrystalline diamond sensor thinned to a thickness of 37 ± 10 µm by the Applied Diamond Corporation (Wilmington, DE, USA). Similar to the sensor used in the Sydor M405V assembly, the sensor was plated at the CINT facility with 25 nm-thick, 3.5 mm × 3.5 mm platinum electrodes. Both substrates used in these studies made use of Element Six Electronic Grade Single Crystal diamond, with nitro­gen contamination of less than 5 p.p.b. Crossed-polarizer studies performed prior to fabrication confirmed an absence of electrically active defects in the region of the sensors exposed to the XPP X-ray beam.

**Figure 2:**
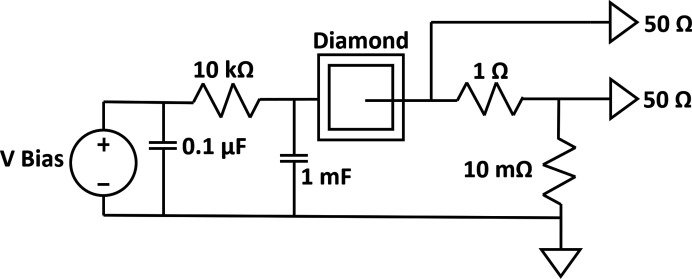
Schematic of the SCIPP assembly readout path; all components except the sensor bias supply are incorporated into the PCB that houses the sensor.

**Figure 3:**
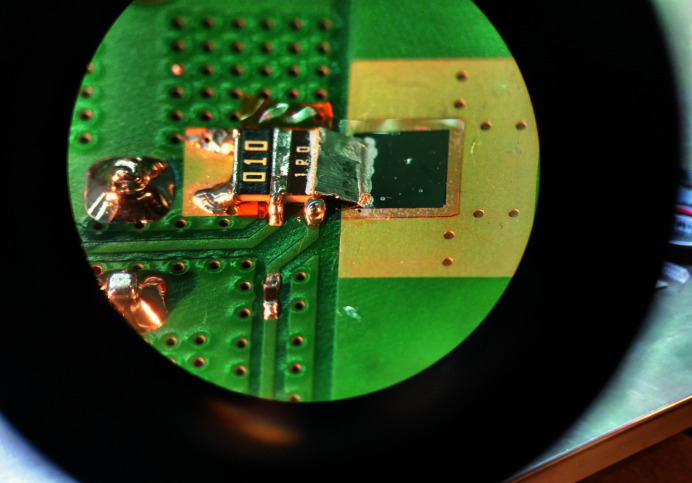
Detail of the readout PCB signal path of the SCIPP assembly, showing the 37 µm-thick diamond sensor, the low-impedance indium band connecting the sensor to the readout network, the series array of 1 Ω and 10 mΩ resistors that shunt the signal to ground, and the two 50 Ω pick-off traces.

**Figure 4:**
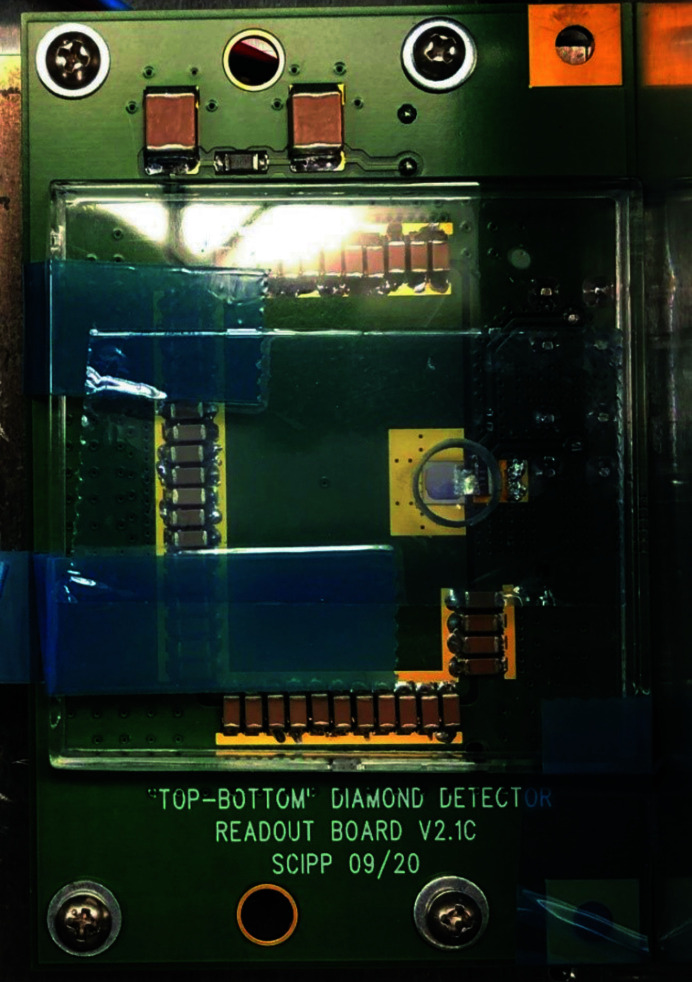
Full view of the SCIPP assembly showing the AC return path provided by a highly parallelized array of 22 µF capacitors. Note that in this photograph the assembly is rotated 180° relative to that of Fig. 3 ▸. The detail shown in Fig. 3 ▸ is located underneath the hole in the protective plexiglass cover.

Fig. 2 ▸ provides a schematic of this ‘SCIPP assembly’, and Fig. 3 ▸ provides a close-up photograph showing the details of its PCB signal path, including the loaded diamond sensor described above. To reduce inductive load associated with bond wires, the sensor is connected to the readout path through a metallic band composed of indium. This band carries signal charge to a series array of two resistors – a 1 Ω resistor followed by a 10 mΩ resistor, with contacts on the long side to minimize inductance – that shunt the signal current directly to ground. 50 Ω pick-off traces make contact with the sensor side of both the 1 Ω and the 10 mΩ resistors, each of which terminates at an SMA connector close to the pickoff point, providing signals than can be digitized and recorded with a high-bandwidth digital storage oscilloscope. Fig. 4 ▸ provides a larger scale view of the SCIPP assembly, showing the signal-trace paths and SMA connector footprints. The AC signal return path is provided by a bank of 44 parallel 22 µF capacitors, amounting to a total capacitance of approximately 1 mF, between ground and the bias plane onto which the sensor is attached making use of Leitsilber Conductive Silver Cement. The redundant parallel paths reduce the overall inductance of the signal return path, while the large capacitance provides an ample reservoir of charge to support the large signal charges generated by the intense XFEL pulse.

Note that the charge collection speed can be very fast for the sensor described above (shown in Fig. 3 ▸). With a saturated drift speed in diamond of approximately 200 µm ns^−1^, absent effects from space-charge and electronic impedance, the nominal charge collection time for the 37 µm-thick sensor is less than 200 ps.

## 3. Data accumulation

The data used in this study were accumulated in two separate runs on the XPP beamline of the LCLS. The commercial (Sydor) sensor assembly was exposed to the XPP beam, tuned to an X-ray energy of 9.83 keV, in the late evening and early morning of 14–15 November 2020. The SCIPP assembly was exposed to the XPP beam, tuned to an energy of 11.89 keV, in the early morning of 6 April, 2021. In each case, the monochromated beam provided pulses that ranged in intensity between 1 µJ and 80 µJ. The data were accumulated with both the full beam as well as with a beam attenuated by 90% through the insertion of a physical attenuator upstream of the sensor assembly.

For the November 2020 running, the signal from the Sydor detector was run directly through the input of an Acqiris digitizer with a sampling rate of 4 Gs s^−1^ and a digitization window of 500 ns. For the April 2021 running, the two signal pick-offs (1 Ω and 10 mΩ) of the SCIPP assembly were read out by a WaveMaster 25 GHz digital storage oscilloscope operating with a sampling rate of 40 Gs s^−1^ and a digitization window of 150 ns. For this latter period, the beam was provided in both unfocused (FWHM estimated to be 350 µm) and focused (FWHM estimated to be 43 µm) modes. For both running periods, high-bandwidth signal-path attenuation was used, as needed, to ensure that the pulses did not saturate the dynamic range of the digitizer and oscilloscope. For the Sydor detector assembly, data were taken with a sensor bias of 500 V, whereas for the SCIPP assembly, data were taken with a sensor bias voltage ranging from 5.4 V to 100 V, although the results reported here only make use of bias voltages of 20 V or greater. For each configuration of beam intensity, beam focus and bias voltage, runs of approximately 1000 pulses were accumulated. For the SCIPP assembly, signals from the 10 mΩ signal pick-off were found to be too noisy to use for the characterization of the detector response, and will not be made use of in the results that follow.

**Figure 5:**
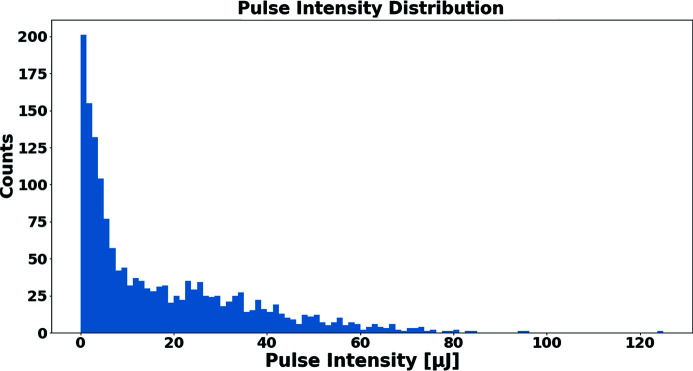
Typical distribution of pulse intensities delivered during the LCLS running, prior to attenuation (if used).

Fig. 5 ▸ shows a typical distribution of pulse intensities delivered during the LCLS running, including running with both unattenuated and 90%-attenuated beam. As described by Feng *et al.* (2011 ▸), pulse intensity measurements made use of the detection of back-scattered X-rays from a partially transmissive thin target using a quadrant X-ray diode array. The system was calibrated against calorimetric and X-ray gas monitor systems as described by Song *et al.* (2019 ▸).

Making use of the energy attenuation coefficients from the US National Institute of Standards and Technology (Hubbell & Seltzer, 2004 ▸), it is estimated that approximately 33% of the XPP X-ray beam was absorbed in the 500 µm-thick sensor mounted in the Sydor assembly, whereas 1–1.5% of the beam was absorbed in the thinner SCIPP assembly sensor.

## 4. Analysis and results

**Figure 6:**
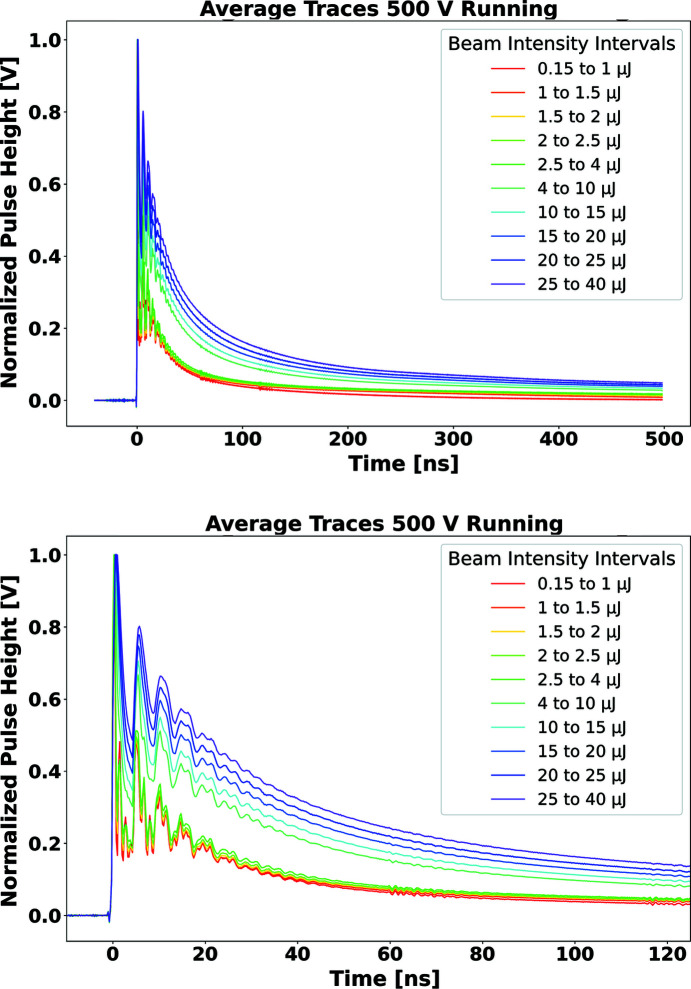
Average temporal signal profiles observed with the Sydor assembly, in bins of delivered pulse intensity. The top plot shows the full 500 ns digitization window; the bottom plot shows the pulse behaviour over the first 125 ns of the excitation, for the purpose of comparing with similar results from the SCIPP assembly (Fig. 7 ▸, top). To compare the temporal properties of the signal current, each pulse is normalized to the same maximum pulse height, which is arbitrarily set to a value of 1.

Fig. 6 ▸ shows the average temporal signal profiles observed with the Sydor assembly, in bins of delivered pulse intensity. Although the nominal collection time is expected to be approximately 2.5 ns for the 500 µm-thick diamond sensor used in the assembly, significant structure is observed over 10–20 ns even for the smallest delivered intensity. In addition, for pulse intensity above 1 µJ, the signal develops a tail that extends beyond the range of the 500 ns digitization window. The presence of this tail outside the digitization window makes it difficult to assess the asymptotic (infinite collection time) linearity of the Sydor assembly.

The behaviour observed for this commercial system suggests that it would not be appropriate for applications requiring sampling rates in the Gs s^−1^ range. The source of the observed structure, and long tail, could be associated with either or both of the impedance of the signal path and the internal charge collection properties of the diamond sensor. In turn, effects caused by degradation of the charge collection properties could be associated with either the large amount of overall generated signal charge or the density of the electron–hole plasma created by the essentially instantaneous absorption of beam quanta.

The SCIPP assembly provides an opportunity to gain further insight into these contributions to the signal degradation, and to potentially also explore the limiting behaviour of diamond sensors for pass-through diagnostics of intense beams. As discussed above, the SCIPP assembly was designed to present a minimal impedance to the diamond sensor signal. However, the assembly was run with two different focal sizes for the beam, leading to a comparison for which the generated charge was identical but the electron–hole plasma density differed roughly by a factor of 30. Observed differences in the properties of the signal-charge collection between unfocused and focused beams could thus only be accounted for by internal processes of the diamond sensor.

**Figure 7:**
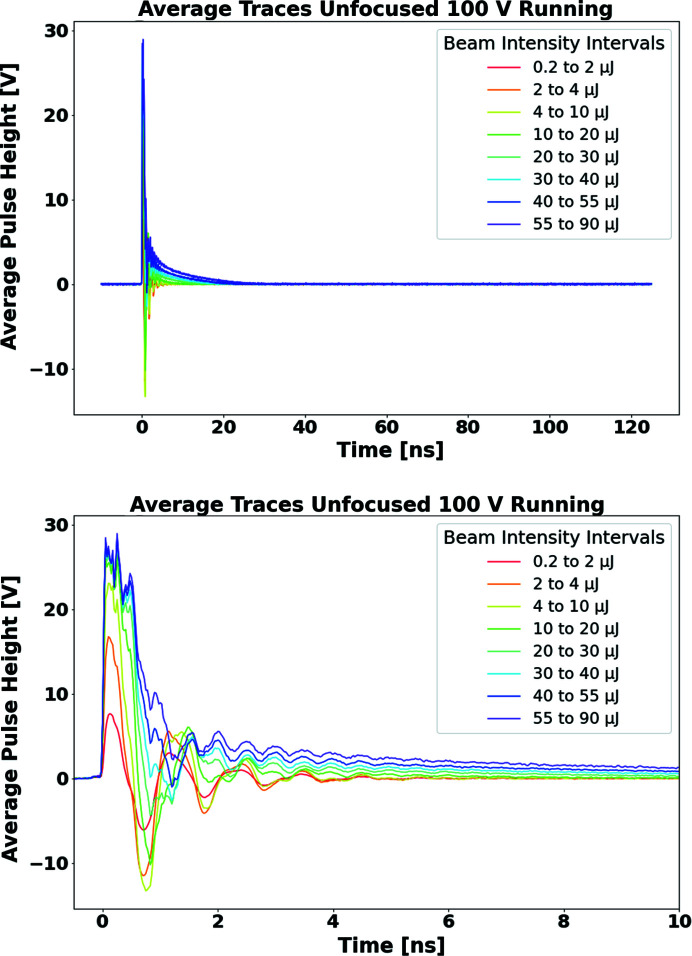
Average temporal signal profiles observed with the SCIPP assembly, in bins of delivered pulse intensity. The top plot shows the full 150 ns digitization window (for ease of comparison with the signal from the Sydor assembly); the bottom plot shows the pulse behaviour over the first 10 ns of the excitation. To maintain a signal at the oscilloscope digitizer within the dynamic range 0–1 V, RF attenuators of known value were added, as needed, to the signal path. Here, pulse heights are adjusted to account for signal-path attenuation, so the vertical axis represents the magnitude of the signal pulse across the shunt resistance.

Fig. 7 ▸ shows the temporal signal profiles for the unfocused beam running of the SCIPP assembly, again in bins of delivered pulse intensity. The signals are significantly faster than those of the Sydor assembly, and, while long tails develop with increasing pulse intensity, all but a small fraction of the charge is collected within the digitization window, allowing for an assessment of the linearity (charge collection efficiency) across the full range of delivered pulse intensity.

The SCIPP assembly signal profiles were analysed as follows. Charge collection current was estimated according to

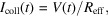

where *I*_coll_ is the estimated charge-collection current, *V* is the measured signal voltage (after accounting for signal-path attenuation as described in the caption of Fig. 7 ▸) and *R*_eff_ is the effective resistance of the signal path, including both the shunt resistance and the 50 Ω termination resistance of the oscilloscope. For the 1 Ω signal pickoff, this parallel combination of 1.01 Ω and 50 Ω led to an effective signal-path resistance of *R*_eff_ = 0.99 Ω.

Total collected charge, as a function of collection time, was estimated by integrating, for signals within a given pulse-energy bin, the average charge collection current from the time of passage of the beam through the sensor (*t* = 0) to the specified collection time,

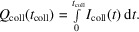

**Figure 8:**
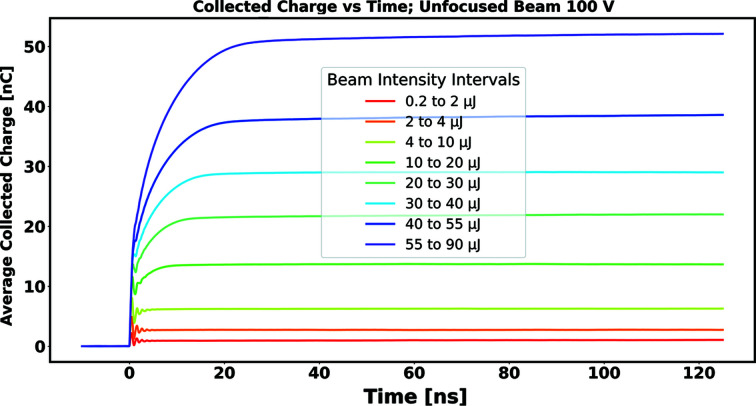
Collected charge as a function of collection time for a sensor bias of 100 V, with the beam focused to a FWHM of 350 µm.

Figs. 8 ▸ and 9 ▸ show the collected charge as a function of time, estimated in this way, for signals arising from successive bins in delivered pulse energy for unfocused and focused beams, respectively. The signals shown are those arising when the diamond sensor was biased to 100 V. In comparing the detector response between the cases of unfocused and focused beams, note that, in a given pulse energy bin, the total delivered charge is essentially identical; what differs between the two cases is the density of generated charge carriers (electron–hole pairs) created inside the diamond bulk, hereafter referred to as the ‘plasma density’ (ρ_P_).

We observed that the time required for full collection of the signal charge increases with total delivered charge, but also is significantly larger when, for a given pulse energy, the beam is focused. This suggests that characteristics of the charge collection, such as collection time and collection efficiency, may be characterized in terms of plasma density, an internal property of the diamond sensor itself, independent of the particular scheme employed to extract its signal.

In the following, we estimate plasma density ρ_P_ according to

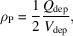

where *Q*_dep_ is the total number of electron–hole pairs generated in the diamond bulk and *V*_dep_ = π*T*(*d*/2)^2^ is the volume occupied by the plasma, with *T* being the sensor thickness and *d* equal to the FWHM quoted above for the unfocused and focused beams (350 µm and 43 µm, respectively). The factor of 1/2 represents the fraction of a two-dimensional Gaussian beam contained within its FWHM. Owing to uncertainties in the measurement of the sensor thickness, and in the knowledge of the lateral size of the XPP beam, the plasma density estimate, defined in this way, has an overall scale uncertainty of approximately ±30%.

The value of *Q*_dep_ in each pulse-energy bin is estimated by multiplying the mean delivered pulse energy in the given bin by a conversion factor of 0.956 nC µJ^−1^. This factor is given by the ratio of the average value of *Q*_coll_ (*t* → ∞), the asymptotic value of the observed collected charge estimate, to the mean delivered pulse energy, for running with a bias voltage of 100 V and a plasma density below 10^16^ cm^−3^, for which the charge collection efficiency is assumed to be 100%. This assumption will be justified below. Note that by making use of compiled attenuation coefficients (Hubbell & Seltzer, 2004 ▸) and accounting for an air column of 45 cm between the XPP beamline vacuum flange and the detector assembly, an expected conversion factor of 0.98 ± 0.26 nC µJ^−1^ can be derived for the 37 ± 10 µm-thick sensor, consistent with the observed value of 0.956 nC µJ^−1^.

Making use of this conversion factor, and the expression for plasma density, both the generated charge and the associated plasma density can be estimated for any delivered pulse energy and for any configuration of the experimental setup. By comparing *Q*_coll_ (*t* → ∞) with the generated charge estimate (the estimate of the number of electron–hole pairs generated by the beam passage), an estimate of the charge collection efficiency can be made as a function of plasma density. Making use of full-energy and 90%-attenuated data accumulated with an unfocused beam, and full-energy data accumulated with a focused beam, the range of plasma density generated in the sensor varied over nearly four orders of magnitude.

**Figure 10:**
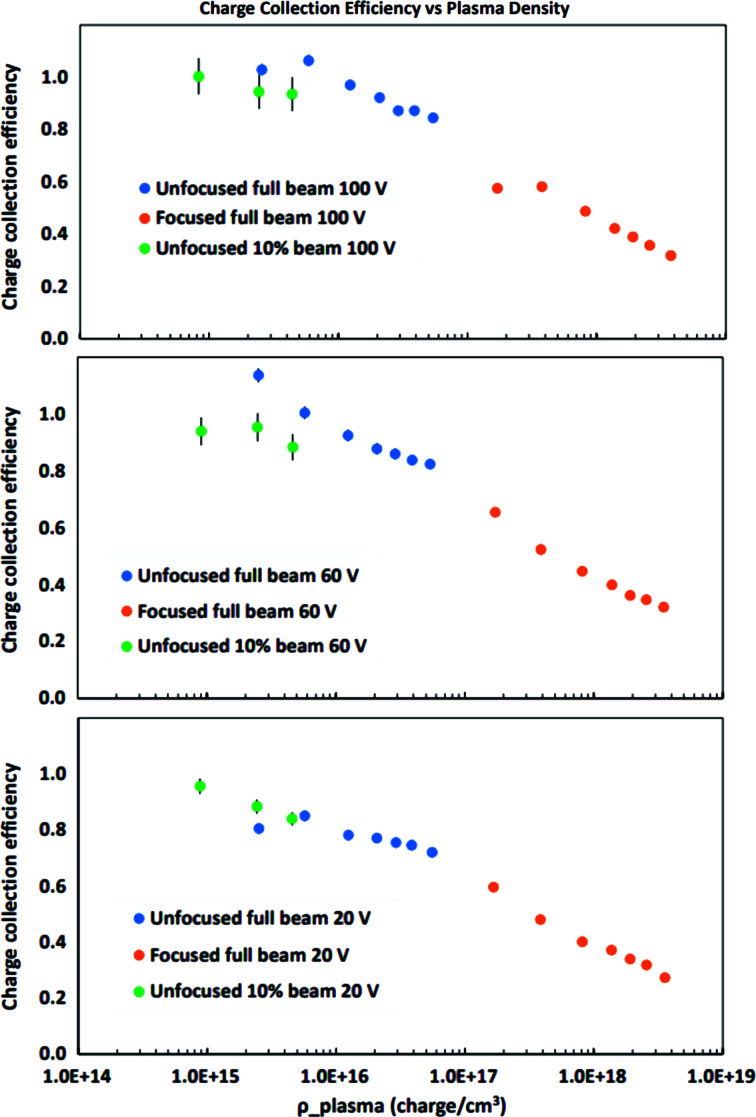
Estimated charge collection efficiency as a function of plasma density for 100 V, 60 V and 20 V sensor bias (bias fields of 2.7 V µm^−1^, 1.6 V µm^−1^ and 0.54 V µm^−1^, respectively).

Fig. 10 ▸ shows the estimated charge collection efficiency as a function of plasma density for the three bias voltages for which data were accumulated for both unfocused and focused beams: 20 V, 60 V and 100 V. For the 100 V running, the consistency of the estimated charge collection efficiency, albeit with limited statistics, for the 90%-attenuated running – at and below the plasma density associated with the lowest energy bin for the cases of unfocused and unattenuated beams – further supports the assumption that the charge collection efficiency is near 100% for that data. For this highest bias voltage (100 V, corresponding to a bias field of approximately 2.7 V µm^−1^), charge collection efficiency loss is observed to occur for plasma densities above 10^16^ charges per cm^3^, with the charge collection efficiency degrading for lesser sensor bias (60 V and 20 V, with bias fields of 1.6 V µm^−1^ and 0.54 V µm^−1^, respectively).

**Figure 11:**
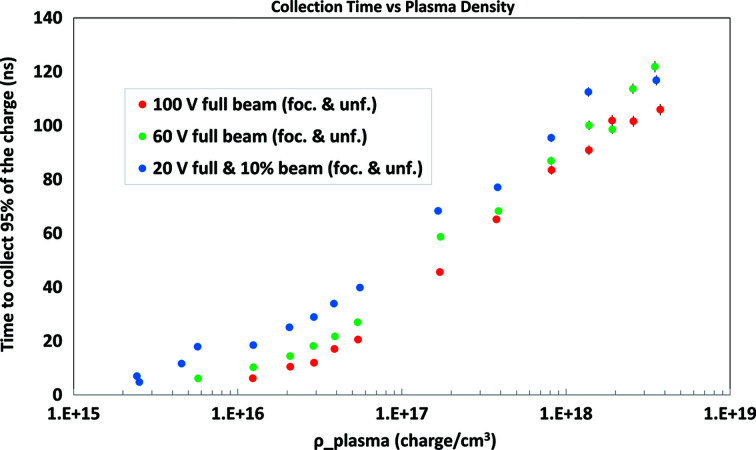
Time required to collect 95% *Q*_coll_ (*t* → ∞) as a function of plasma density, for various detector-biasing levels.

From the information shown in Figs. 8 ▸ and 9 ▸, as well as corresponding information for running with sensor biases of 20 V and 60 V, the time required to accumulate a given fraction of the asymptotic value *Q*_coll_ (*t* → ∞) can be estimated as a function of plasma density. Values of this estimate for a fraction of 95% of *Q*_coll_(*t* → ∞) are displayed in Fig. 11 ▸ as a function of plasma density. The charge collection time, characterized in this way, depends strongly on both plasma density and applied bias field, and approaches 100 ns even for the highest bias field (2.7 V µm^−1^) explored in this study.

## 5. Summary and conclusions

Making use of intense X-ray beams from the XPP beamline of the LCLS, a study was performed to explore the suitability of the use of pure, low-defect diamond sensors for a high frame-rate pass-through diagnostic for high-intensity X-ray beams. The best results were obtained with an apparatus custom-designed to have a low-impedance signal path. Within this apparatus, a thin (37 ± 10 µm) diamond sensor, biased to fields as high as 2.7 V µm^−1^, was exposed to 11.89 keV X-ray pulses with intensities of up to 80 µJ. For this highest bias field, charge collection efficiency was found to be maintained for plasma densities as high as 10^16^ charges per cm^3^, with the charge collection efficiency improving monotonically with applied bias voltage. Charge collection time, characterized by the amount of time required to accumulate 95% of the asymptotic value of collected charge, was also found to depend strongly on plasma density and detector bias voltage. Though the results suggest that charge collection speed and efficiency may be improved by increasing the bias field above 2.7 V µm^−1^ (the maximum value used in the study), it seems that the intrinsic charge collection properties of monocrystalline diamond will present challenges to the development of pass-through diagnostics for high-intensity XFEL beams (which can approach several millijoules), especially for high-repetition-rate applications. This work has suggested a number of studies that can be carried out to further improve the response of the readout path, with the goal of producing a diagnostic with multi-GHz frame-rate capabilities. To this end, the authors are currently exploring the use of high-bandwidth RF solvers to model multi-GHz signal transport, and the further compactification of the readout path. In addition, fabrication techniques needed to produce a quadrant diamond sensor with electrode gaps on the order of 10 µm, required for position sensing, are also under development.

## Figures and Tables

**Figure 9:**
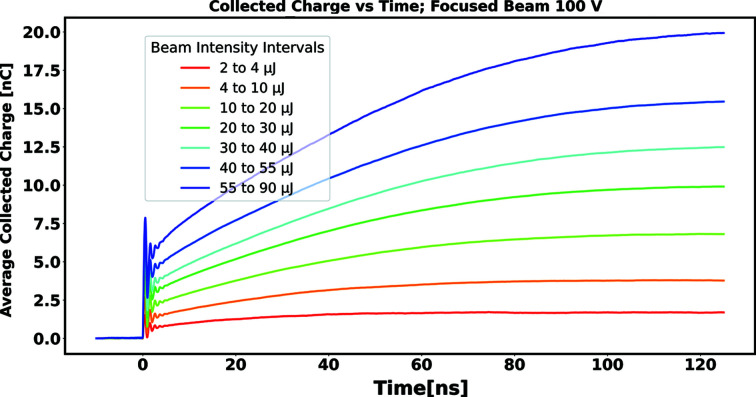
Collected charge as a function of collection time for a sensor bias of 100 V, with the beam focused to a FWHM of 43 µm.
